# Preparation, Thermal, and Thermo-Mechanical Characterization of Polymeric Blends Based on Di(meth)acrylate Monomers

**DOI:** 10.3390/polym13060878

**Published:** 2021-03-12

**Authors:** Krystyna Wnuczek, Andrzej Puszka, Łukasz Klapiszewski, Beata Podkościelna

**Affiliations:** 1 Department of Polymer Chemistry, Institute of Chemical Sciences, Faculty of Chemistry, Maria Curie-Skłodowska University, M. Curie-Skłodowska Sq.3., 20-031 Lublin, Poland; andrzej.puszka@umcs.pl (A.P.); beatapod@umcs.pl (B.P.); 2 Faculty of Chemical Technology, Institute of Chemical Technology and Engineering, Poznań University of Technology, Berdychewo 4, PL-60965 Poznań, Poland; lukasz.klapiszewski@put.poznan.pl

**Keywords:** polycarbonate, polymeric blends, acrylates

## Abstract

This study presents the preparation and the thermo-mechanical characteristics of polymeric blends based on di(meth)acrylates monomers. Bisphenol A glycerolate diacrylate (BPA.GDA) or ethylene glycol dimethacrylate (EGDMA) were used as crosslinking monomers. Methyl methacrylate (MMA) was used as an active solvent in both copolymerization approaches. Commercial polycarbonate (PC) was used as a modifying soluble additive. The preparation of blends and method of polymerization by using UV initiator (Irqacure^®^ 651) was proposed. Two parallel sets of MMA-based materials were obtained. The first included more harmless linear hydrocarbons (EGDMA + MMA), whereas the second included the usually used aromatic copolymers (BPA.GDA + MMA). The influence of different amounts of PC on the physicochemical properties was discussed in detail. Chemical structures of the copolymers were confirmed by attenuated total reflection–Fourier transform infrared (ATR/FT-IR) spectroscopy. Thermo-mechanical properties of the synthesized materials were investigated by means of differential scanning calorimetry (DSC), thermogravimetric (TG/DTG) analyses, and dynamic mechanical analysis (DMA). The hardness of the obtained materials was also tested. In order to evaluate the surface of the materials, their images were obtained with the use of atomic force microscopy (AFM).

## 1. Introduction

The science of polymers is currently undergoing rapid development, both in the field of basic research regarding known polymers and new polymeric materials. The development of new methods of synthesis focused on the production of materials with improved properties as well as extending the current area of polymer application are crucial priorities.

Polymer blends belong to a class of materials in which at least two polymers (or polymer and monomer) are blended together to create a macroscopically homogeneous material with different physical properties. The basic goal of the production of such materials is to obtain a product with more favorable chemical and physical properties than the polymers included in the blend [1]. The following features which make polymer blends increasingly attractive can be distinguished: The possibility of obtaining a cheap material with a strictly designed set of properties, the possibility of extending the application area of polymers existing on the market, the possibility of improving processing and reducing production waste [2,3,4,5]. Polymer blends have found application both in everyday life, industry and advanced technologies. There has been a lot of progress in the field of blends in recent years [6,7,8,9]. They have been developed to meet specific technical requirements, such as: Better process ability, greater impact strength, greater resistance to deformation at elevated temperature, and better resistance to physicochemical factors. The properties of the main components are therefore changed by controlled admixing [10,11,12,13].

Preparation of polymer blends is relatively simple. There are many ways to obtain polymer blends, the most common of which is the mixing of solid polymers above the yield point. Due to the specificity of the conducted process, plastic mixing can be carried out using continuous and periodic methods. In some cases, extruders are used [14,15,16]. The final polymeric material could be modified by addition of small amounts of other components to achieve the desired properties. All ingredients could be mixed at various ratios to produces new blends with significantly different properties [17,18].

Blends based on poly(methylmethacrylate) (PMMA) and polycarbonate (PC) have been found in the literature, however the number of reports which present the synthesis of blends based on ethyleneglycol dimethacrylate with methyl methacrylate (EGDMA + MMA) and a PC filler are limited. Although PMMA exhibits a fragile mechanical behavior, mixing it with PC results in polymer blends with good mechanical performance. Debier et al. synthesized blends by melting PC and PMMA in THF and proposed a reaction mechanism between the polymers. This mechanism takes into account the influence of the degradation of the components under the experimental conditions in which the chemical reaction was observed [19]. In another study, imide units were copolymerized with MMA in order to improve the compatibility between PC and acrylics through specific interactions or internal repulsion [20]. Moussaif et al. obtained a PMMA-g-PC copolymer that could act as a compatibilizer for the PC/PVDF blend [21]. Kunar et al. melt blended bisphenol-A polycarbonate and poly(butylene terephthalate) with ethylene-n-butylacrylate-glycidylmethacrylate terpolymer at various proportions in order to study the effects of compatibilizers on mechanical, thermal, and flow properties of the blends [22]. The studies concerning the PMMA/PC blend were reported by Macedo et al. [23] The effect of processing conditions on the physicochemical properties of poly(methyl methacrylate)/polycarbonate blends was considered. The obtained polymer blends were characterized by a synergistic combination of mechanical properties of polycarbonate and polymethylmethacrylate.

Abtahi et al. reported obtaining π-conjugated polymer blends with revised thermoelectric power factors. Blending π-conjugated polymers provides a possibility of manipulating charge transport properties and improving the performance of organic thermoelectrics [24]. The conjugated polymer blends were also synthesized by Savagian et al. The article presents how the composition of dioxythiophene-based electrochromic polymers (ECPs) blends can be adapted to access a range of black hues with complete coverage of the visible spectrum [25]. In turn, Jiang et al. presented self-assembled structures of blended random and block copolymers to tune the encapsulation and release of lipophilic cargo molecules [26].

Polycarbonates (PC) have been widely applied in automotive industries, construction, electronics, and preparation of medical equipment due to their specific mechanical, thermal, and optical properties [27,28]. Many industrial sectors have used polycarbonates in view of their excellent physical properties such as transparency, high toughness, high percent elongation, high impact fact strength and high thermal stability [29,30,31,32,33,34,35,36]. Many important application areas of polycarbonates include the manufacturing of household products (CD production, lighting, polycarbonate panels) [34,35,36,37]. Developing new types of polymeric materials and their characterizations allows to expand the range of applications of polycarbonates in various fields of the economy.

In the present study, new materials in the form of polymeric blends with the addition of commercial polycarbonate were prepared and characterized. The methyl methacrylate (MMA) was used as an active solvent, because it is characterized by low toxicity, and is appropriate for high viscosity monomers. We attempted to develop a synthesis method of PC-polymeric blends with improved physicochemical properties in comparison with poly(methyl methacrylate) (PMMA). The polymeric materials were obtained by using the photopolymerization method which has many advantages: Low temperature of the process, high speed, low cost and low energy. In addition, a more harmless monomers system based on linear hydrocarbons (EGDMA + MMA) in comparison with the commonly used the aromatic monomer BPA.GDA was also applied. Thermal and thermo-mechanical properties of both obtained systems were compared by means TG, DSC, and DMA analyses. Additionally, spectroscopic characterization (ATR-FTIR), hardness, and swelling properties of the new polymeric blends were evaluated.

## 2. Materials and Methods

### 2.1. Materials

**Figure 1:**
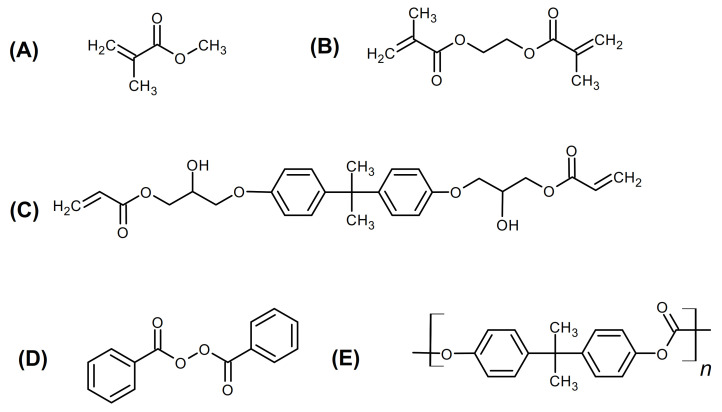
Chemical structures of the reagents: Methyl methacrylate (MMA) (**A**); ethylene glycol dimethacrylate (EGDMA) 5 (**B**); bisphenol A glycerol diacrylate (**C**); 2,2-dimethoxy-2-phenylaceto-phenone (**D**), and commercial polycarbonate (PC) (**E**).

Bisphenol A glycerolate (1 glycerol/phenol) diacrylate (BPA.GDA) (Sigma Aldrich, Steinheim am Albuch, Germany) and ethylene glycol dimethacrylate (EGDMA, ≥97.5%) (Sigma Aldrich, Steinheim am Albuch, Germany) were used as monomers in the polymerization reaction. Moreover, 2,2-dimethoxy-2-phenylaceto-phenone (Irqacure^®^ 651) was applied (Sigma Aldrich, Steinheim am Albuch, Germany) as the photoinitiator of polymerization. In both systems, methyl methacrylate (MMA, ≥99.0%) (Merck, Darm-stadt, Germany) was employed as an active solvent. The PC (polycarbonate) was pur-chased from Lotte Chemical (Seoul, South Korea), density 1.15–1.25 g/cm^3^, molecular weight >1.000 g/mol. Methane dichloride (99.8%) (Merck, Darmstadt, Germany) was used as a solvent for commercial PC. Structures of listed reagents are presented in Figure 1. For swelling test, we used the acetone (99.5%) and hydrochloric acid (36.5–38.0%) from Merck. The purified water was provided by Millipore UMCS (Lublin, Poland).

### 2.2. Methods

The attenuated total reflection (ATR) was recorded based on Fourier transform infrared (ATR/FT-IR) spectroscopy using a TENSOR 27 Bruker (Bruker GmbH, Mannheim, Germany) spectrometer equipped with a diamond crystal (Ettlingen, Germany). The spectra were recorded in the range of 4000–600 cm^−1^ with 32 scans per spectrum at a resolution of 4 cm^−1^.

Thermogravimetric analysis TG/DTG was conducted using a STA 449 Jupiter F1, Netzsch (Selb, Germany). The samples were heated from 25 to 600 °C at a rate of 10 °C/min in a dynamic atmosphere of helium (25 cm^3^/min). An empty Al_2_O_3_ crucible was used as a reference. The thermal stability factors, such as loss mass temperatures (T_5%_, T_10%_,T_50%_), as well as temperatures of maximum mass loss (T_max_) and residual mass (RM) were estimated.

Differential scanning calorimetry (DSC) curves were obtained with the use of a DSC Netzsch 204 calorimeter Netzsch (Günzbung, Germany). The measurements were taken in the aluminum pans with a pierced lid with the sample mass was approx. 10 mg under nitrogen atmosphere (30 cm^3^/min). Dynamic scans were performed at a heating rate of 10 °C/min in the temperature range 0–200 °C. An empty aluminum crucible was used as reference.

DMA measurements were performed by means of dynamic mechanical analyzer (DMA) Q 800 TA Instruments (New Castle, DE, USA) using the dual cantilever clamp. Measurements for all samples were conducted in the temperature range of −50–180 °C at a constant heating rate of 3 °C/min with a constant frequency of 1 Hz. Samples with dimensions equal to 65 mm × 10 mm × 2 mm were tested. Viscoelastic properties of the obtained materials were estimated based on the changes of storage modulus (E’), loss modulus (E’’) as well as the changes of tan delta at constant frequency depending on temperature. The T_g_ was identified as the maximum of the tan delta. Based on the tan delta curves, the full-width at half maximum (FWHM) was also determined.

The hardness of the materials was measured based on the Shore D method using a 7206/H04 analog hardness testing apparatus (Zwick, Ulm, Germany) at 20 °C. Readings were taken after 15 s.

The images of samples were obtained using an atomic force microscopy (AFM)—Analytical Laboratory, Faculty of Chemistry, UMCS, Lublin, Poland). All measurements in the tapping operation modes were carried out using a NanoScope V AFM (Veeco, New York City, NY, USA) equipped with the NanoScope 8.10 software (Bruker Corporation, Germany). A rectangular Si cantilever/tip (Veeco, New York City, NY, USA) with a spring constant of 20–80 N/m and resonance frequency of 300 kHz was used. The resolution of the scans obtained was equal to 256 × 256 pixels. The topography and peak force error images were obtained simultaneously. The data were analyzed using the Nanoscope Analysis ver. 1.40 software (Veeco, New York City, NY, USA).

### 2.3. Preparation of Blends

**Figure 2:**
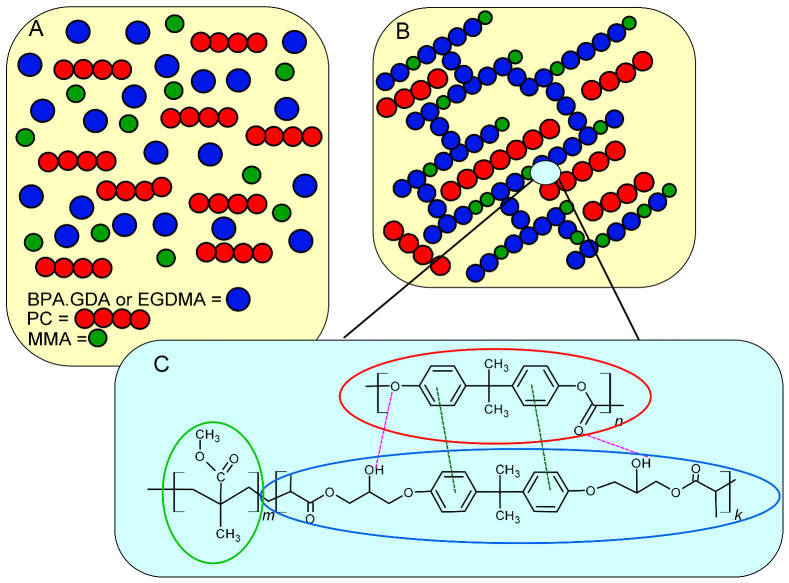
Proposed scheme of polymeric blends structure—before (**A**) and after (**B**) the polymerization reaction with potential interactions between the functional groups (**C**).

The BPA.GDA + MMA + PC and EGDMA + MMA + PC blends were prepared using the photopolymerization technique. An appropriate amount of active solvent MMA (methyl methacrylate) was added to the monomer (BPA.GDA or EGDMA) at the wt.% ratio of 3:7. The monomers were stirred at room temperature until a homogenous solution was obtained. Then, the PC solutions were carefully introduced. After addition, the polymer (PC) was dissolved in CH_2_Cl_2_ (2 h, room temperature). The whole content was stirred to obtain a homogeneous mixture and next put into the oven in order to evaporate the solvent (for 20 min in 30 °C). Finally, the calculated amount of UV initiator (2% *w/w*) (Irqacure^®^ 651) was added to the sample. The contents of the beaker was poured into glass molds (10 mm × 8 mm × 2 mm) and polymerized under a UV lamp for 40 min. The samples were heated at 80 °C for 30 min after taking them out from under UV lamps. The detailed information regarding reagents and their amounts is presented in Table 1. The proposed scheme of polymeric blends structure is presented in Figure 2.

As can be seen, there are possible interactions among groups present in molecules. The chemical hydrogen bonds are created between, e.g., carbonyl groups (C=O) and hydroxyl groups (–OH) from BPA.GDA linear aliphatic fragments. Additionally, π-π interactions between the aromatic rings of benzene present in both PC and BPA.GDA chains are also possible. The interactions of BPA.GDA chains with PC should be much stronger than in case of EGDMA-PC, in which the above mentioned groups are not present. As confirmed our measurements, polymer blends based on EGDMA are more brittle and less resistant, which may be due to weaker interactions between the polymer chains.

## 3. Results and Discussion

A mixture of monomers (MMA and EGDMA or BPA.GDA), and PC was exposed to UV radiation in the presence of the photoinitiator and yielded 8 blends which possessed the same thickness. The obtained materials were cut and subjected to the physicochemical tests.

### 3.1. Structural Characterization of PC Blends Using ATR/FT-IR

**Figure 3:**
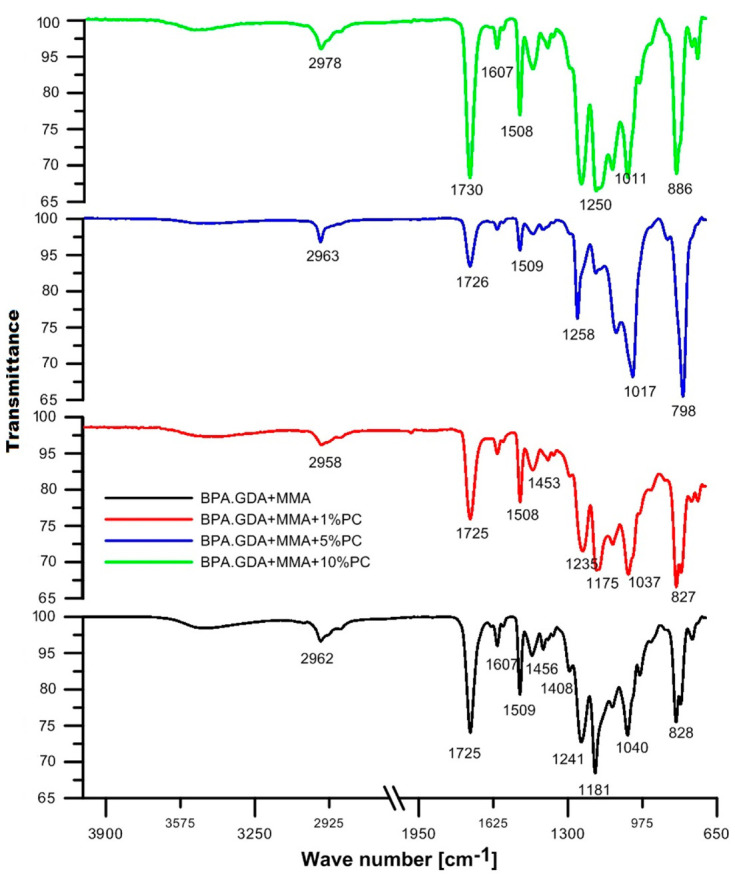
Attenuated total reflection–Fourier transform infrared (ATR/FT-IR) spectra of bisphenol A glycerolate diacrylate (BPA.GDA) + MMA derived blend.

**Figure 4:**
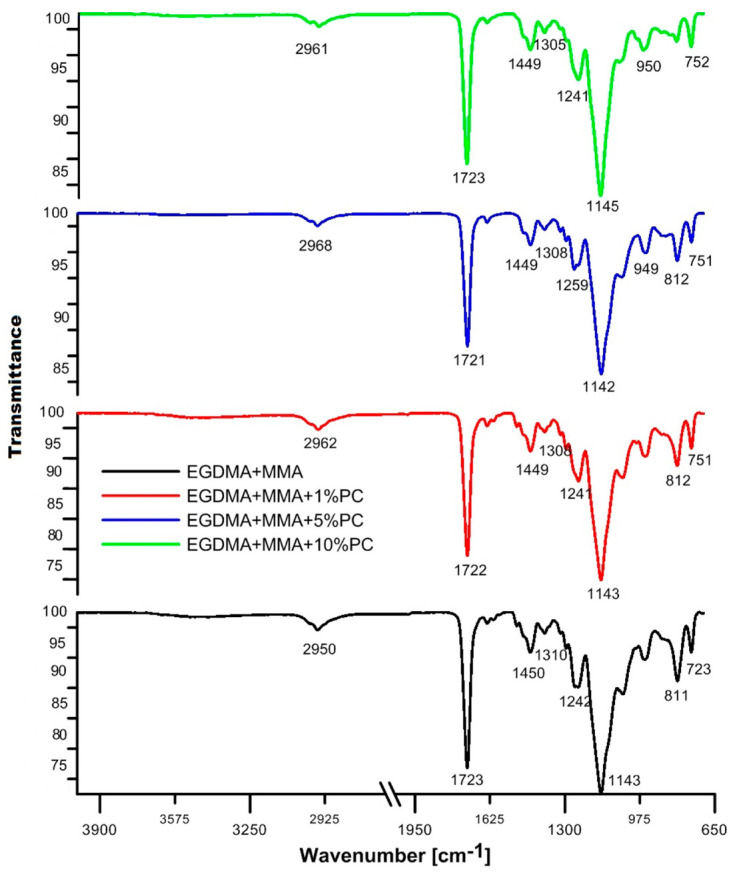
ATR/FT-IR spectra of EGDMA + MMA derived blends.

The ATR/FT-IR results are shown in Figure 3 and Figure 4. In Figure 3, the ATR/FT-IR spectra of BPA.GDA + MMA blends are visible. Generally, the spectra of all obtained systems are characterized by a similar course. There is a clear signal at 3490 cm^−1^ associated with the hydroxyl group. The broad absorption band in the spectrum indicates the presence of –OH groups from the BPA.GDA monomer. In the modified blends, this signal becomes weaker. With the addition of PC, signals from this part of the blend decrease. This is due to the aromatic structure that originates from the PC. Another clear signal at approx. 2900 cm^−1^ comes from the aliphatic part of the C-H groups. It is visible for all materials. The observed peaks in the range of 2985–2962 cm^−1^ correspond to C–H stretching vibrations in the methylene groups. Their intensities are similar for all PC blends. The signal from the carbonyl group is in the range of 1730–1725 cm^−1^ for all materials. The peak at 1508 cm^−1^ is associated with the stretching vibrations of –C=C in benzene rings and aromatic skeletal vibrations. These signals are at a similar level of intensity, showing that the aromatic systems of PC were incorporated into the blends. The peaks at approx. 1455–1453 cm^−1^ originate from C–H deformation in the –CH_2_ and –CH_3_ groups. The multiple signals in the range of 1250–1011 cm^−1^ were due to the C–O stretching in C–O–CH_3_, whereas the peak at approx. 886–797 cm^−1^ was characteristic for 1,4-substituted aromatic rings. In summary, the addition of PC does not drastically change the course of the ATR/FT-IR curves of the copolymers, however, it does affect the intensity of the signals. The exact values of the wavenumbers are given in Tables S1 and S2 in the Supplementary Materials.

The ATR/FT-IR spectra of EGDMA + MMA blends are presented in Figure 4. The copolymer EGDMA + MMA + 5% PC shows the highest absorbance intensity. The rest of the spectra are practically identical. When comparing the BPA.GDA + MMA spectrum with EGDMA + MMA, vibrations originating from C–H aliphatic –CH_2_ and –CH_3_ groups, C=O signals and multiple signals in the range of 1310–1142 cm^−1^ are visible in both spectra. Characteristic bands at approx. 1240 cm^−1^ of the functional group C–O are visible on the curves. The typical vibrations for the ring are also visible.

The addition of polycarbonate to the blends resulted in changed intensity of the band originating from the carbonyl group, C–C bond and the aromatic ring structure in the BPA.GDA + MMA spectrum. In the case of the EGDMA + MMA blend, all four spectra are very similar. The polycarbonate added to the material included two-positioned rings in the para position in its structure. This is evidenced by vibrations in the range of 800–860 cm^−1^. However, it should be noted that in the case of blends based on bisphenol A, this effect also originates from the BPA.GDA compound. All monomers and polycarbonates contained an ester bond in their structure, therefore each spectrum shows a characteristic signal in the range of 1720–1750 cm^−1^.

### 3.2. Thermogravimetric Analysis

**Figure 5:**
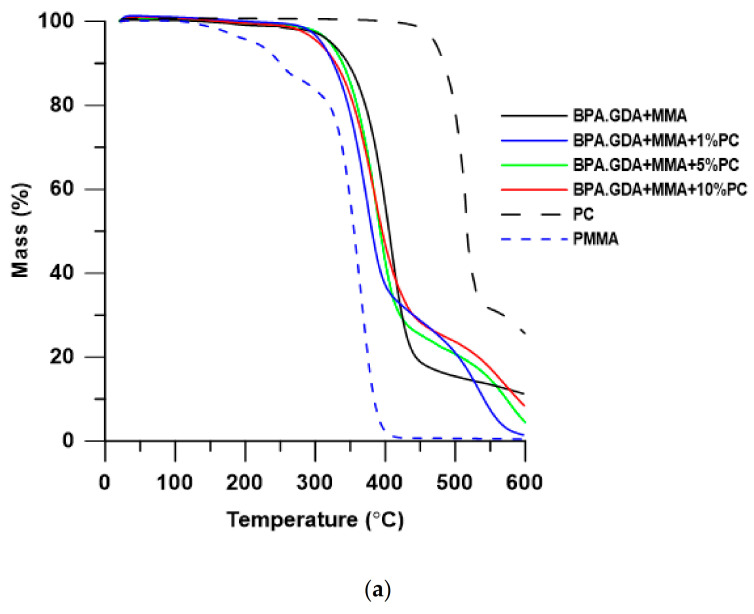

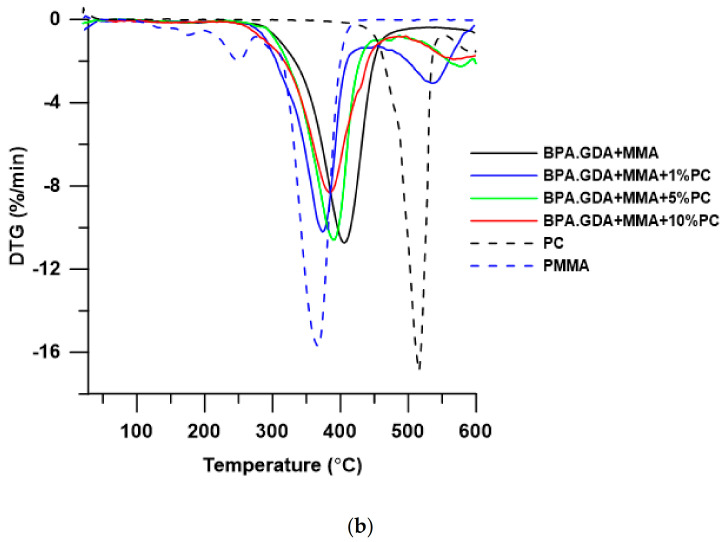
TG (**a**) and DTG (**b**) curves of BPA.GDA + MMA based blends.

**Figure 6:**
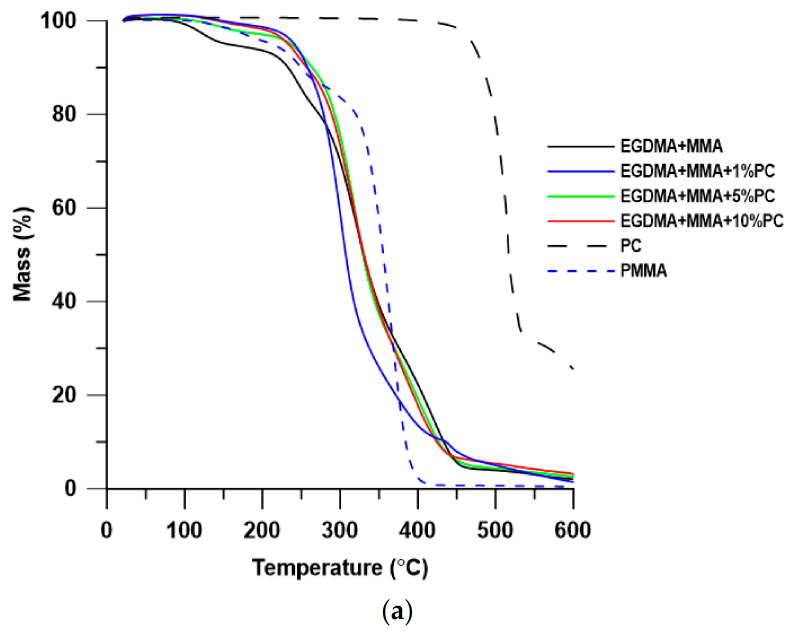

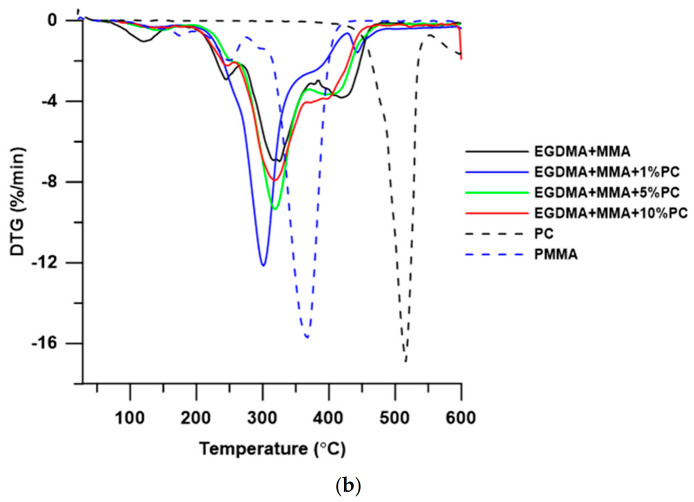
TG (**a**) and DTG (**b**) curves of EGDMA + MMA-based blends.

Thermal stability and degradation behavior of the obtained blends were investigated by means of thermogravimetry. The TG/DTG results of thermal decomposition process in the inert atmosphere of helium are presented in Table 2 and Figure 5 and Figure 6. For a more detailed analysis of the materials, the results of the thermogravimetric analysis for pure PC and PMMA were also performed.

As is well known, the thermal stability of PMMA is relatively low, its decomposition starts below 200 °C. The use of BPA.GDA + MMA mixture increases the thermal stability of the material. It is due to the presence of aromatic rings in BPA.GDA having higher dissociation energy. In turn, addition of PC to the polymer (and thus increase of the amount of aromatic rings in the material) also increases the thermal stability of the EGDMA-based materials. In the case of BPA.GDA-based blends, this relationship was not observed.

As can be seen in Table 2, blends based on BGA.GDA exhibit better thermal stability than those with EGDMA and their T_5%_ is over 300 °C. This is due to the chemical structure of these materials and is consistent with the theory. In both series of blends with the addition of PC, the residual mass in the measuring cup increased with the increase of the percentage of PC, with generally more amounts of deposit being formed in the series of blends based on BPA.GDA.

As can be seen based on the shape of the DTG curves, the thermal decomposition of the obtained blends occurs in several stages. In the case of BPA.GDA-based materials, thermal decomposition takes place in two steps. The first stage (T_max_ in the range 374–391 °C corresponding to 73–75% of mass loss) is probably responsible for the distribution of fragments of the material composed of MMA and BPA.GDA, while the second peak (T_max_ in the range of 536–567 °C, corresponding to 16–26% of mass loss) is responsible mainly for the decomposition of the PC contained in the obtained blends. With the increasing addition of PC, the intensity of the peaks in the DTG curve at T_max_ was reduced, while an increasing amount of solid precipitates is observed after decomposition (except materials without PC). It is difficult to provide a clear explanation for the results, since the distribution of multi-component materials, including the polymer blend, strongly influences the interfacial specific interactions.

For the EGDMA + MMA-based blends, the TG curves are characterized by a similar course. Nevertheless, there are significant differences in the onset of decomposition of polymeric blends. The addition of PC has a positive effect on the thermal resistance of materials. In the case of EGDMA + MMA polymer, the loss of 5% mass is at 157 °C, after the addition of even 1 wt.% of PC the temperature increases up to 241 °C. In all cases, the TG curve shows no clear mass loss related to the decomposition of the PC addition to the matrix. When analyzing the shape of TG and DTG curves, the decomposition of EGDMA-MMA blends proceeded in several stages. The first stage (T_max_ in the range of 121–137 °C corresponding to 2–3% of mass loss) may be related to the evaporation of residual solvents, moisture and unreacted monomers [38,39]. Subsequent maxima of the rate of mass loss (i.e., T_max_ = 245–258 °C with 7–12% of mass loss and T_max_ = 301–320 °C with 55–64% of mass loss) are probably responsible for the degradation of EGDMA, while the maximum rate of mass loss at T_max_ (temperature range of 396–442 °C and 19–27% of mass loss) is related to the distribution of the fragments derived from MMA.

**Figure 7:**
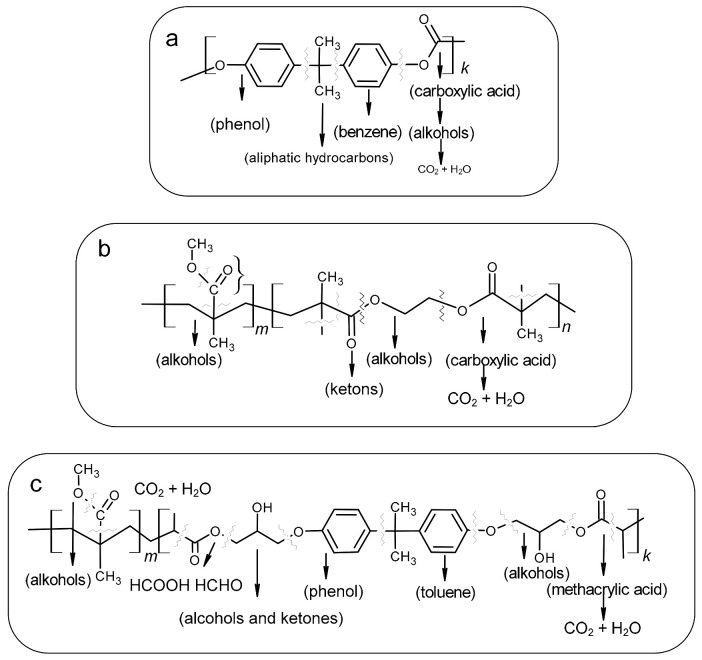
Proposed mechanisms of polymer fragmentation under heating: (**a**) polycarbonate, (**b**) EGDMA-MMA copolymers, (**c**) BPA.GDA-MMA copolymers.

In summary, the improved thermal stability was observed for the BPA.GDA blends and not the corresponding blends of EGDMA, which is strictly connected with the chemical structure (presence of aromatic rings) of these compounds and their thermal stability. In order to analyze the course of the decomposition of the obtained materials, determine the evolved gaseous products and propose the degradation mechanism, it would be necessary to perform a more precise and, at the same time, more complicated coupled analysis, e.g., TG/FT-IR/MS. The proposed mechanisms of polymer network fragmentation for all studied materials are presented in Figure 7. The suggested mechanism is based on our earlier research, thermal decomposition of BPA.GDA-derived copolymers and literature data [39,40,41]. In the case of polycarbonates, their decomposition is very similar to BPA.GDA due to the presence the same structural fragments (Bisphenol A) and leads to aromatic compounds such as phenol, toluene and benzene, as well as small aliphatic hydrocarbons, alcohols, ketones, and then H_2_O and CO_2_. In case of EGDMA-MMA-based copolymers, the main products of thermal decomposition (apart from carbon dioxide and water) included small alcohols, ketones, and methacrylic acid.

### 3.3. Differential Scanning Calorimetry Characterization

**Figure 8:**
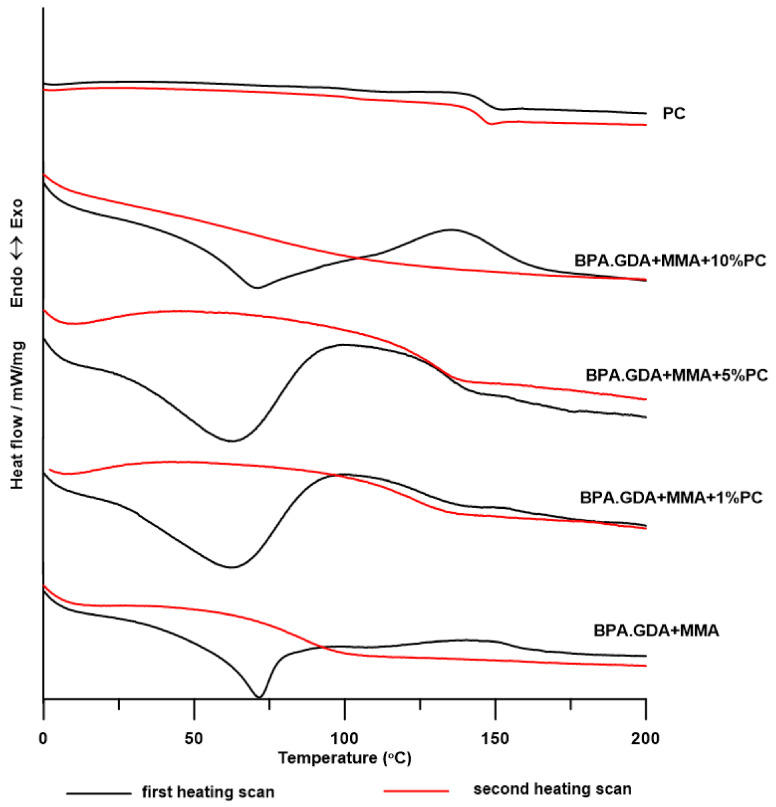
DSC curves of BPA.GDA + MMA blends.

**Figure 9:**
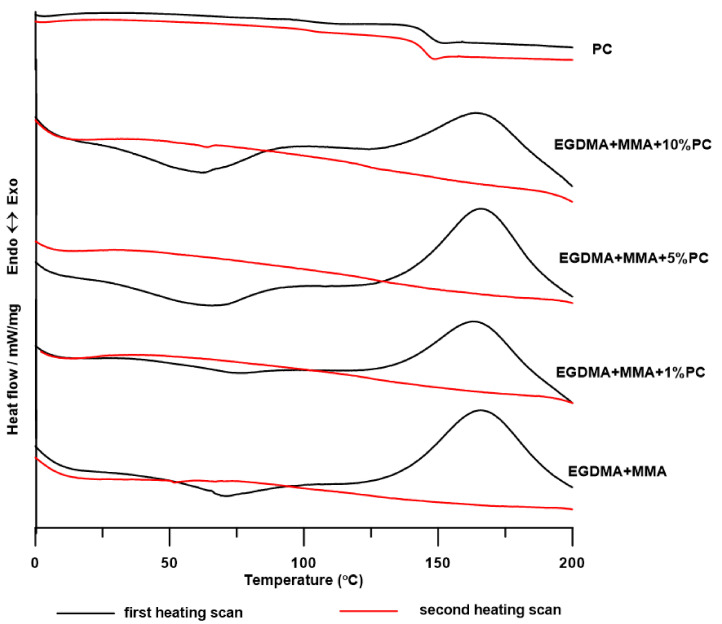
DSC curves for EGDMA + MMA blends.

DSC measurements were carried out in the course of two heating cycles at temperatures ranging from 0 to 200 °C. The numerical data of the DSC analysis is presented in Table 3, while the DSC curves of the obtained materials are shown in Figure 8 and Figure 9.

As can be seen in both graphs, the DSC curves from the first heating cycle reveal both endo- and exothermic changes. Endothermic peaks (with T_m_ in the range of 62–72 °C) are responsible for the melting of the crystalline form of PMMA, the abnormal formation of which for blends with 30 wt.% PMMA content has already been noticed by Faria and Moreira [42]. This conversion is irreversible, no crystallization of PMMA occurs during cooling and no crystallite melting peaks are observed in the second course of heating. The heat capacity of these transformations for the BPA.GDA blend series decreases with the increasing amount of PC in the material, while for the EGDMA blends the trend is reversed. As shown in the literature, the T_g_ of pure PMMA ranges from 104 to 122 °C and depends on the molar mass of the obtained polymer [38,42,43], while the T_m_ of the PMMA is approximately 72 °C [44]. The broad exothermic peaks (in fact it is a change in slope of curve that comes from an endothermic peak with T_recryst_ at 101 and 107 °C) for BPA.GDA + MMA + 1% PC and BPA.GDA + MMA + 5% PC blends are responsible for the recrystallization of PMMA crystallites or with polymorphic changes in the crystal structure. The exothermic peaks for other materials (BPA.GDA + MMA and BPA.GDA + MMA + 10% PC) at T_p_ in the range of 136–166 °C are responsible for the cross-linking of materials. The energy effects of cross-linking EGDMA-based blends are higher than those of BPA.GDA and it decreases with increasing PC content in the material. This may be due to the fact that the increasing number of PC chains causes the material to become more rigid, so access to unreacted methacrylic groups is limited. The DSC curves from the second heating do not reveal exo- and endothermic changes (which indicates that the materials are amorphous), while the glass transitions can be clearly seen only for blends BPA.GDA MMA + 1% PC, BPA.GDA + MMA + 5% PC. DSC analysis performed for PC showed that it was amorphous material (on the DSC curves there is no exo- and endothermic peaks) characterized by a T_g_ of 146 °C.

### 3.4. DMA Test

**Figure 10:**
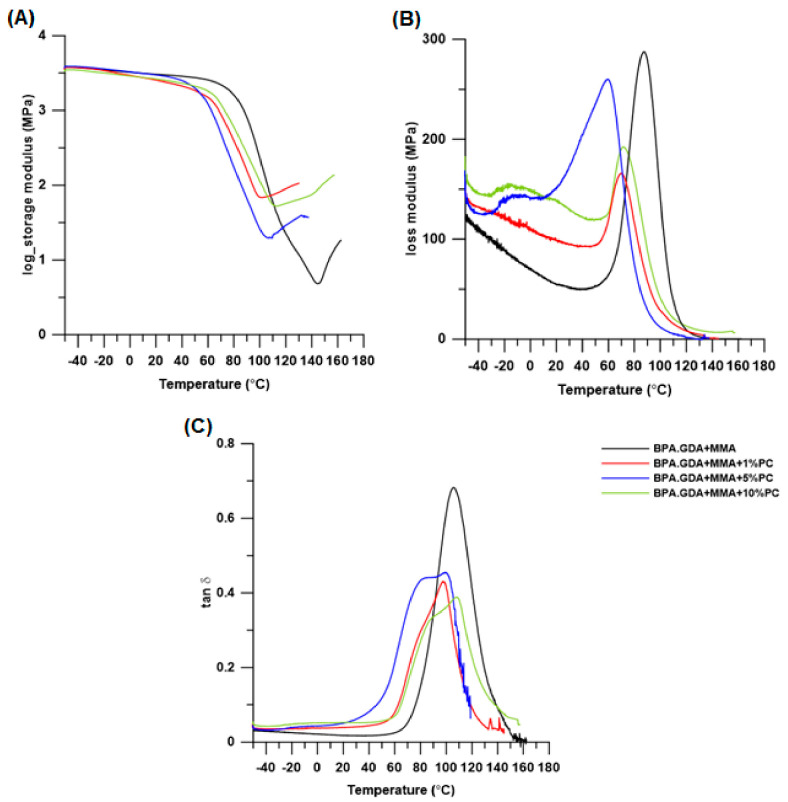
DMA curves for BPA.GDA + MMA blends: Storage modulus (E’) (**A**), loss modulus (E’’) (**B**) and damping factor (tan δ) (**C**).

In order to complete the DSC analysis and to better define the phase changes occurring in the obtained materials during heating, the DMA analysis was performed, and its results are presented in Table 4 and Figure 10. Unfortunately, it was not possible to obtain blends based on EGDMA in such sizes to prepare samples for DMA analysis (these materials had numerous cracks, they were very brittle, which can be seen in the photos of samples in Swelling test chapter.

As shown in Figure 10A, the loss modulus curve (E’) drops sharply with increasing temperature in the glass transition, and then its increase is visible. It results from the process of cross-linking of unreacted monomers (PMMA and BPA.GDA). The cross-linking process was also noticed during the DSC analysis (exothermic peaks in the temperature range 136–166 °C). There is no visible flattening of the E’ curve, which determines the highly elastic state of the tested material, that should be visible in the case of linear polymers. The loss modulus curves (Figure 10B) indicate fairly good homogeneity of the materials in the glass transition region, with peak maxima indicating that the materials differed significantly in strengths and damping properties. The worst damping properties (and the highest values of peak value of tan delta and loss modulus) were observed in case of the comparative material without PC. Comparing the shape of the tan delta curves of the tested blends, it can be seen that the obtained materials were not homogeneous. The FWHM value of the tan delta peak is the parameter which allows to determine the homogeneity of the tested sample [45,46,47,48,49]. The FWHM values were much higher compared to the starting material. Moreover, these peaks were not symmetrical and two glass transition temperatures could be observed (except for the starting material BPA.GDA + MMA). This indicates that the miscibility of the components in the blends was unsatisfactory [50]. The miscibility of PC and PMMA has been confirmed in many studies in which the authors found that these polymers are fully compatible [51,52,53]. The T_g_ values determined based on tan delta correspond to the glass transition of PMMA. Due to the fact that the sample was damaged during the analysis in the temperature range of 140–150 °C as a result of the polymerization process of unreacted monomers (T_p_ values from DSC analysis), it was not possible to determine the T_g_ for the pure PC component of our blend (Figure S1, Supplementary Materials).

### 3.5. Hardness

**Figure 11:**
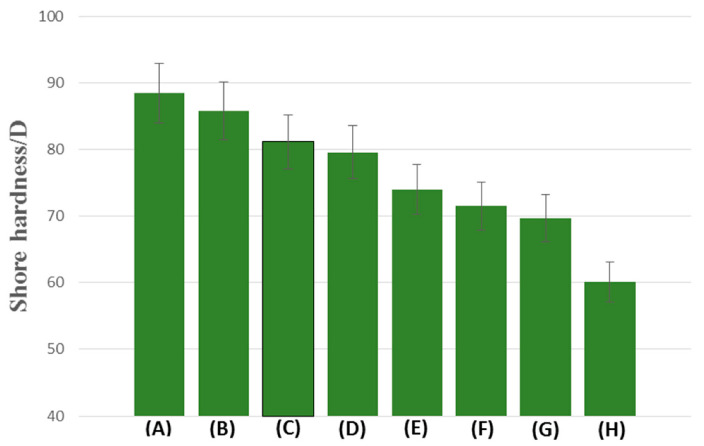
The Shore hardness of: EGDMA + MMA (**A**), EGDMA + MMA + 1% PC (B), EGDMA + MMA + 5% PC (**C**), EGDMA + MMA + 10% PC (**D**), BPA.GDA + MMA (**E**), BPA.GDA + MMA + 1% PC (**F**), BPA.GDA + MMA + 5% PC (**G**) and BPA.GDA + MMA + 10% PC (**H**).

The principle of this measurement is based on measuring the depth of indenter penetration into the material of the studied polymer samples on a scale from 0 to 100 °Sh. The results of the study are shown in Figure 11. The EGDMA + MMA polymer (86 °Sh) was characterized by the highest value of hardness, while the lowest value was observed in case of BPA.GDA + MMA + 10% PC (60 °Sh). The materials based on EGDMA monomer possess a more rigid structure, susceptible to cracking and for this reason the values of hardness data are higher. The blends containing BPA.GDA had a lower Shore hardness values in the range 60–73 °Sh. Generally, the addition of PC lowered the hardness and the polymeric blends became more flexible. The exact values of the hardness are given in Table S3 in the Supplementary Materials.

Goliszek et al. [54] described the effect of the addition of lignin on the composite based on BPA.GDA + EHA. The highest Shore-D hardness values were obtained for biocomposites with the addition of 2.5 and 5% lignin. After applying 20% lignin, a decrease of the hardness value was observed. Compared to our blend, the addition of polycarbonate lowered the hardness. The highest value of Shore hardness was observed for the EGDMA + MMA blend (86 °Sh). Table S7 summarizes the properties of the polymer blends.

### 3.6. Swelling Tests

**Figure 12:**
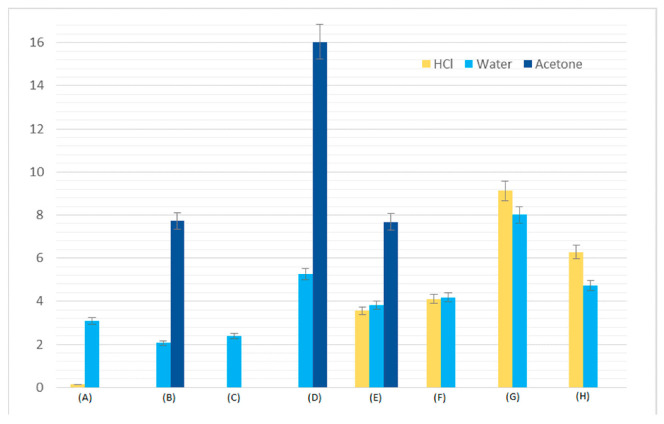
Swelling charts. (**A**) BPA + MMA, (**B**) BPA + MMA +1 %PC, (**C**) BPA + MMA + 5%PC, (**D**) BPA + MMA + 10%PC, (**E**) EGDMA + MMA, (**F**) EGDMA + MMA + 1%PC, (**G**) EGDMA + MMA + 5%PC, (**H**) EGDMA + MMA + 10%PC.

**Figure 13:**
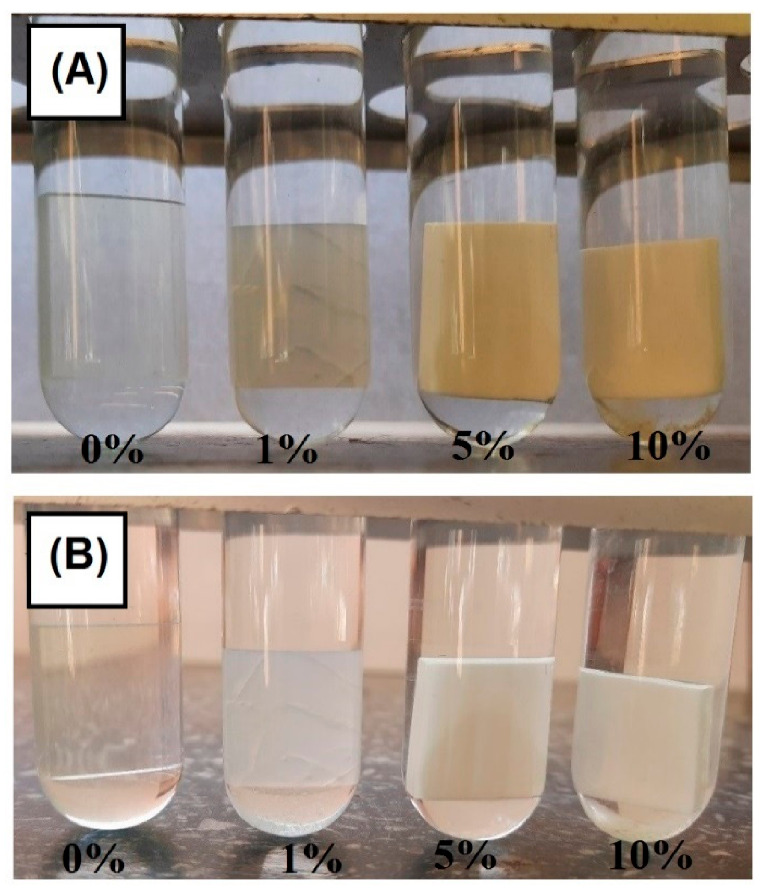
Swelling test for EGDMA + MMA-based materials in acetone (**A**) and BPA.GDA + MMA-based materials in HCl (**B**).

Swellability is a factor that defines accessibility of the internal chemical structure in the cross-linked polymers for penetration by solvent molecules. This factor gives information on how the resulting materials will behave under varying environmental conditions (different solvents). The swellability tests in hydrochloric acid, water and acetone were carried out. The results are summarized in Figure 12. (The exact values of all swelling tests are given in Tables S4–S6 in the Supplementary Materials). Based on the results presented above, it can be concluded that the greatest weight changes were obtained for hydrochloric acid. Water has a significantly lower swelling capacity. In turn, the blends turned out to be not resistant to acetone. In the case of BPA.GDA +MMA blends treated with hydrochloric acid, there was a weight loss in the final phase of the test. Most likely, the acid was destroying the exterior of these materials. Only the copolymer with 1% of PC proved to be resistant. EGDMA + MMA-based copolymers showed greater resistance to the acid. The amount of PC in the materials did not significantly change the acid resistance. The increase in PC content in the material during tests with water caused a slight increase in swelling. Certainly, the destruction of blends after prolonged contact with acetone was influenced by the inhomogeneity of the surface which greatly accelerated the penetration of solvent into the polymer blends. Swelling process is shown in Figure 13.

### 3.7. Images of Obtained Blends—AFM Test

**Figure 14:**
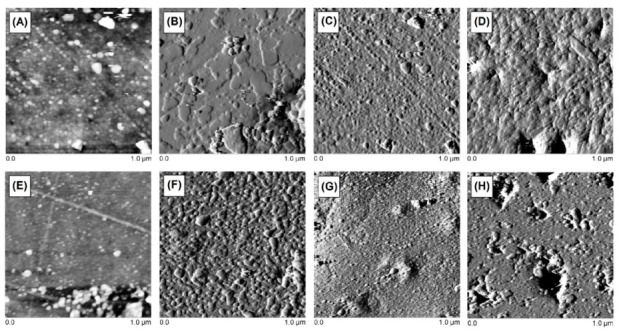
AFM photos of obtained blends: BPA.GDA + MMA (**A**), BPA.GDA + MMA + 1% PC (**B**), BPA.GDA + MMA + 5% PC (**C**), BPA.GDA + MMA + 10% PC (**D**), EGDMA + MMA (**E**), EGDMA + MMA + 1% PC (**F**), EGDMA + MMA + 5% PC (**G**) and EGDMA + MMA + 10% PC (**H**).

The image of the fragments of polymeric blend obtained using AFM is illustrated in Figure 14. All samples exhibited a similar structure. The blends are a macroscopically homogeneous mixture. Under changes of the temperature, various fragments of polymers may behave differently and hence the differences during the DMA analysis were observed. Based on the images, it can be established that all ingredients of the blends are uniformly distributed (particularly at the macroscopic level) [55]. In case of the largest ratio of polycarbonate (10 wt.%), some small changes in the surface structure can be seen, which may be the reason for the phase separation. Based on these AFM images, it can be concluded that the surface roughness of the composite increases with the inclusion of more PC in the composite structure.

## 4. Conclusions

New blends based on methyl methacrylate (MMA) and bisphenol A glycerolate (1 glycerol/phenol) diacrylate (BPA.GDA) or ethylene glycol dimethacrylate (EGDMA) with the additives commercial polycarbonate were successfully obtained during UV photopolymerization. The structure of polymeric blends as well as parent copolymers were studied by ATR/FT-IR spectroscopy. The DSC data confirmed that all of materials are amorphous, whereas the endothermic peaks (T_m_ in the range of 62–72 °C) are responsible for the melting of the crystalline form of PMMA. Based on the thermogravimetric analysis, it can be stated that the addition of PC to the blends increased its thermal resistance (especially visible in the case of EGDMA + MMA based blends). DMA analysis showed that the BPA.GDA-based blends indicated some heterogeneity but were characterized by better damping properties in comparison to the starting material. The EGDMA + MMA based blends demonstrated higher hardness (80–86 °Sh) compared to analogous materials based on BPA.GDA + MMA (60–74 °Sh) but an increase in the brittleness of the EGDMA system was observed. The addition of PC decreased the hardness of the materials. According to swelling tests, the amount of PC affects the resistance to solvents. Our materials proved to be sensitive to acetone in the swelling test. For BPA.GDA + MMA + 10% PC, the highest values of the swelling factor were recorded. Images obtained using the AFM test indicated that the obtained blends were characterized by a similar homogenous structure with small differences in surface structure.

## Figures and Tables

**Table 1:** Amounts of components used for the synthesis.

Sample	BPA.GDA/EGDMA + MMA	BPA.GDA/EGDMA + MMA + 1% PC	BPA.GDA/EGDMA + MMA + 5% PC	BPA.GDA/EGDMA + MMA + 10% PC
MMA (g)	3	3	3	3
BPA.GDA/EGDMA (g)	7	7	7	7
PC (wt.%)	0	1	5	10
CH_2_Cl_2_ (cm^3^)	0	1	2	4
Initiator (g)	0.200	0.202	0.210	0.220

**Table 2:** Thermogravimetric data of the obtained blends.

Material	T_5%_^a^ (^o^C)	T_10%_^b^ (^o^C)	T_50%_^c^ (^o^C)	T_max_^d^ (^o^C)				RM ^e^ (%)
PC	471	484	516	-	516	-	-	25.58
PMMA ^f^	213	272	355	180	250	367	-	0.40
BPA.GDA + MMA	323	347	406	407	-	-	-	11.12
BPA.GDA + MMA + 1% PC	309	326	381	374	536	-	-	1.39
BPA.GDA + MMA + 5% PC	321	339	393	391	577	-	-	4.50
BPA.GDA + MMA + 10% PC	304	329	395	384	567	-	-	8.23
EGDMA + MMA	157	233	330	121	245	320	420	2.07
EGDMA + MMA + 1% PC	241	258	307	137	-	301	442	1.44
EGDMA + MMA + 5% PC	237	264	329	137	258	320	396	2.58
EGDMA + MMA + 10% PC	234	257	331	135	245	320	397	3.10

^a, b, c^ The temperature of 5%, 10%, and 50% mass loss from the TGA curve, respectively; ^d^ The temperatures of the maximum rate of mass loss from the derivative TGA (DTG) curves; ^e^ Residual mass at 600 °C; ^f^ TG and DTG data from [38].

**Table 3:** Differential scanning calorimetry (DSC) data of obtained materials.

Sample	T_m_ (°C)	Δ*H_m_* (J/g)	T_recryst_ (°C)	Δ*H_recryst_* (J/g)	T_g_^1^ (°C)	T_g_^2^ (°C)	T_p_ (°C)	Δ*H_p_* (J/g)
PC	-	-	-	-	146	146	-	-
BPA.GDA + MMA	72	11.7	-	-	-	92	140	3.1
BPA.GDA + MMA + 1% PC	62	20.9	101	8.7	127	120	-	-
BPA.GDA + MMA + 5% PC	62	14.1	107	12.1	131	128	-	-
BPA.GDA + MMA + 10% PC	71	15.0	-	-	-	-	136	8.1
EGDMA + MMA	71	3.0	-	-	-	-	166	28.3
EGDMA + MMA + 1% PC	77	2.4	-	-	-	-	163	26.7
EGDMA + MMA + 5% PC	66	6.8	-	-	-	-	166	23.2
EGDMA + MMA + 10% PC	63	7.4	-	-	-	-	164	19.7

Where: ^1,2^—first or second heating scan, respectively; T_m_, T_recryst_, T_p_—temperatures of melting, recrystallization and cross-linking, respectively; ΔH_m_, ΔH_recryst_, ΔH_p—_heat capacities of melting, recrystallization and cross-linking, respectively.

**Table 4:** Dynamic mechanical analysis (DMA) data of obtained materials.

Sample	T_g_ (°C)		tan δ _max_	E’’ (MPa)	FWHM (°C)
	tan δ	E’’			
PC BPA.GDA + MMA	144 105.5	121 87.7	0.16 0.67	57.9 260	37.8 32.6
BPA.GDA+ MMA + 1% PC	97.3	71.1	0.39	100	34.6
BPA.GDA + MMA + 5% PC	~80; 99.4	59.9	0.44	177	47.6
BPA.GDA + MMA + 10% PC	~90; 108.4	72.9	0.33	99	44.9

## Supplementary Materials

The following are available online https://www.mdpi.com/2073-4360/13/6/878/s1. S1. Structural Characterization of PC Blends using ATR/FT-IR, Table S1. Wavenumbers (cm^−1^) of characteristic bands visible on ATR/FT-IR spectra, Table S2. Wavenumbers (cm^−1^) of characteristic bands visible on ATR/FT-IR spectra, S2. Hardness test, the abbreviations used in the Tables: BPA.GDA+MMA (A), BPA.GDA+MMA+1%PC (B), BPA.GDA+MMA+5%PC (C), BPA.GDA+MMA+10%PC (D), EGDMA+MMA (E), EGDMA+MMA+1%PC (F), EGDMA+MMA+5%PC (G), EGDMA+MMA+10%PC (H), Table S3. Hardness measurements (°Sh), S3. Swelling test, The swellability tests in hydrochloric acid, water and acetone were carried out. The results are summarized in Tables S4–S6. The swelling ratio was calculated based on the formula (1), Table S4. Swelling studies in HCl (1 M) solvent, Table S5. Swelling studies in H_2_O, Table S6. Swelling studies in acetone, S5. DMA analysis of PC, Figure S1. DMA analysis of pure PC, S6. Summarizing, Table S7. Summarizing table of some properties.
