# Supplementary material for: Preparation, Thermal, and Thermo-Mechanical Characterization of Polymeric Blends Based on Di(meth)acrylate Monomers
Source: Polymers (Basel). 2021 Mar 12;13(6):878. doi: 10.3390/polym13060878 (PMC7999132; doi:10.3390/polym13060878)
Supplement: Supplementary file 1 [file polymers-13-00878-s001.pdf]

polymers

MDPI

Supplementary Materials

Preparation, thermal and thermo-mechanical characterization of polymeric blends based on di(meth)acrylate monomers

Krystyna Wnuczek[1], Andrzej Puszka[1], Łukasz Klapiszewski[2] and Beata Podkościelna[1]

1 Department of Polymer Chemistry, Institute of Chemical Sciences, Faculty of Chemistry, Maria Curie-Skłodowska University; M. Curie-Skłodowska Sq.3., 20-031 Lublin, Poland; krystyna.wnuczek@poczta.umcs.lublin.pl (K.W.); andrzej.puszka@umcs.pl (A.P.); beatapod@umcs.pl (B.P.)
2 Poznań University of Technology, Faculty of Chemical Technology, Institute of Chemical Technology and Engineering, Berdychewo 4, PL-60965 Poznań, Poland; lukasz.klapiszewski@put.poznan.pl (Ł.K.)
* Correspondence: krystyna.wnuczek@poczta.umcs.lublin.pl (K.W.)

S1. Structural Characterization of PC Blends using ATR/FT-IR

Table S1. Wavenumbers (cm^{-1}) of characteristic bands visible on ATR/FT-IR spectra.

Sample	C–H aliph.	C–H arom.	C=C arom.	C–O	C=O	C-C	-OH
**BPA.GDA+MMA**	2962	1455<br>828	1607<br>1508	1039	1725	1250	3490
**BPA.GDA+MMA+1%PC**	2985	1453<br>827	1508	1175	1725	1260	-
**BPA.GDA+MMA+5%PC**	2963	797	1508	1016	1726	1270	-
**BPA.GDA+MMA+10%PC**	2978	886	1607<br>1508	1011	1730	1250	-

Citation: Lastname, F.; Lastname, F.; Lastname, F. Title. *Polymers* **2021**, *13*, x. https://doi.org/10.3390/polym13060878

Academic Editor: Firstname Lastname
Received: date
Accepted: date
Published: date

Publisher's Note: MDPI stays neutral with regard to jurisdictional claims in published maps and institutional affiliations.

Copyright: © 2021 by the authors. Submitted for possible open access publication under the terms and conditions of the Creative Commons Attribution (CC BY) license (http://creativecommons.org/licenses/by/4.0/).

Table S2. Wavenumbers (cm^{-1}) of characteristic bands visible on ATR/FT-IR spectra.

Sample	C–H aliph.	C–H arom.	C=O	C–C
**EGDMA+MMA**	2950<br>1310<br>1143	1450<br>811	1723	1242<br>723
**EGDMA+MMA+1%PC**	2962<br>1308<br>1143	1449<br>949<br>812	1722	1241<br>751
**EGDMA+MMA+5%PC**	2968<br>1308<br>1142	1449<br>812	1721	1259<br>751
**EGDMA+MMA+10%PC**	2961<br>1305<br>1145	1449<br>950	1723	1241<br>752

S2. Hardness test

The abbreviations used in the Tables: BPA.GDA+MMA (A), BPA.GDA+MMA+1%PC (B), BPA.GDA+MMA+5%PC (C), BPA.GDA+MMA+10%PC (D), EGDMA+MMA (E), EGDMA+MMA+1%PC (F), EGDMA+MMA+5%PC (G), EGDMA+MMA+10%PC (H).

Table S3. Hardness measurements (°Sh).

Material	I	II	III	IV	V	Average
**(A)**	72.5	74.5	73.5	74.5	75.0	74.0
**(B)**	71.0	70.5	71.5	72.0	72.5	71.5
**(C)**	69.5	68.5	70.0	71.0	69.5	69.7
**(D)**	60.5	60.0	59.5	60.5	60.0	60.1
**(E)**	88.0	89.0	88.5	88.5	88.5	88.5
**(F)**	85.5	85.5	86.0	85.5	86.5	85.8
**(G)**	81.0	81.0	81.5	81.0	81.5	81.2
**(H)**	79.5	79.5	80.0	80.0	79.0	79.6

S3. Swelling test

The swellability tests in hydrochloric acid, water and acetone were carried out. The results are summarized in Tables S4-S6. The swelling ratio was calculated based on the formula (1)

$$X = 100\ \%-(\frac{M0}{M28}*100\ \%) \quad (1)$$

Table S4. Swelling studies in HCl (1 M) solvent.

**Test time (day)**	**Weight gain (g)**								**Mass change**
	**0**	**4**	**8**	**12**	**16**	**20**	**24**	**28**	**X**
**(A)**	0.5449	0.5465	0.5470	0.5481	0.5496	0.5472	0.5456	0.5431	**0.13**
**(B)**	0.5681	0.5451	0.5294	0.5321	0.5428	0.5437	0.5482	0.5496	**-3.36**
**(C)**	0.4989	0.4960	0.4924	0.4920	0.4939	0.4930	0.4932	0.4934	**-1.11**
**(D)**	0.4276	0.4250	0.4227	0.4226	0.4226	0.4227	0.4221	0.4223	**-1.26**
**(E)**	0.4306	0.4350	0.4447	0.4445	0.4497	0.4475	0.4465	0.4466	**3.56**
**(F)**	0.4807	0.4860	0.4975	0.5007	0.5019	0.5017	0.5013	0.5014	**4.11**
**(G)**	0.5704	0.5990	0.6194	0.6213	0.6321	0.6325	0.6277	0.6242	**9.13**
**(H)**	0.4502	0.4595	0.4685	0.4699	0.4701	0.4785	0.4803	0.4819	**6.27**

Table S5. Swelling studies in H_2O.

**Test time (day)**	**Weight gain (g)**								**Mass change**
	**0**	**4**	**8**	**12**	**16**	**20**	**24**	**28**	**X**
**(A)**	0.5309	0.5317	0.5328	0.5375	0.5427	0.5455	0.5464	0.5478	**3.09**
**(B)**	0.4602	0.4599	0.4593	0.4681	0.4678	0.4688	0.4695	0.4699	**2.06**
**(C)**	0.4505	0.4550	0.4560	0.4610	0.4634	0.4620	0.4605	0.4615	**2.38**
**(D)**	0.4072	0.4111	0.4123	0.4129	0.4130	0.4234	0.4244	0.4298	**5.26**
**(E)**	0.3991	0.4070	0.4091	0.4154	0.4194	0.4168	0.4158	0.4150	**3.83**
**(F)**	0.4264	0.4355	0.4419	0.4522	0.4597	0.4533	0.4477	0.4450	**4.18**
**(G)**	0.6695	0.6952	0.7183	0.7223	0.7297	0.7240	0.7265	0.7277	**8.00**
**(H)**	0.4137	0.4199	0.4274	0.4789	0.4300	0.4325	0.4334	0.4342	**4.72**

Table S6. Swelling studies in acetone.

**Test time (day)**	**Weight gain (g)**								**Mass change**
	**0**	**4**	**8**	**12**	**16**	**20**	**24**	**28**	**X**
**(A)**	0.5302	0.5706	-	-	-	-	-	-	**-**
**(B)**	0.5310	0.5410	0.5438	0.5494	0.5506	0.5621	0.5713	0.5755	**7.73**
**(C)**	0.4623	0.6214	-	-	-	-	-	-	**-**
**(D)**	0.4518	0.4683	0.4750	0.4913	0.5069	0.5170	0.5274	0.5381	**16.04**
**(E)**	0.4176	0.4356	0.4514	0.4520	0.4537	0.4531	0.4527	0.4523	**7.67**
**(F)**	0.4005	0.4720	0.4940	0.4980	0.4999	-	-	-	**-**
**(G)**	0.5217	0.5318	-	-	-	-	-	-	**-**
**(H)**	0.4675	0.4785	0.4895	0.4899	0.4996	-	-	-	**-**

(-) the material broke down into many pieces

S5. DMA analysis of PC

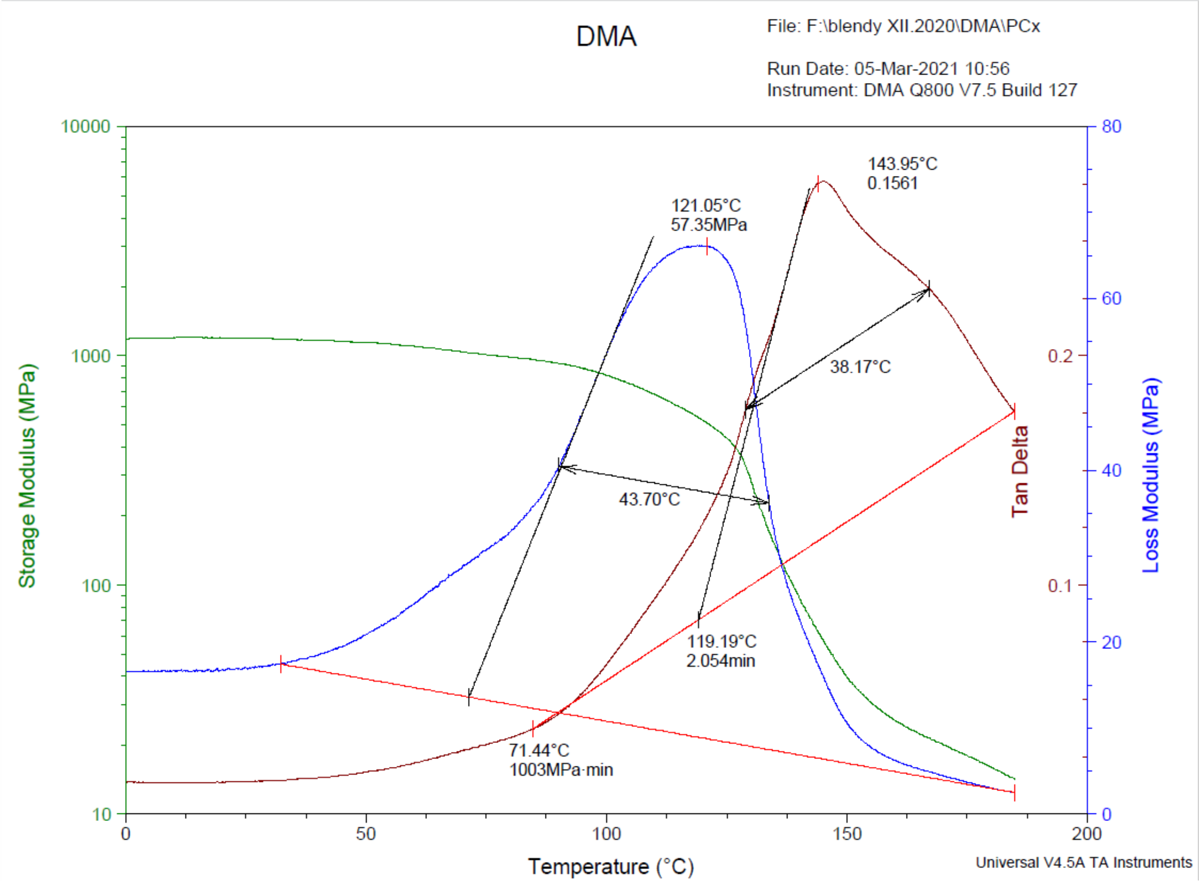

Figure S1. **DMA analysis of pure PC**

S6. Summarizing

Table S7. Summarizing table of some properties.

	BPA.GDA+ MMA	BPA.GDA+ MMA+PC	EGDMA+MMA	EGDMA+ MMA+PC	PMMA
**$T_{10\%}$ (°C)**	347	326-339	233	257-264	277
**tan δ (°C)**	105.5	97.3	-	-	-
**FWHM (°C)**	32.6	34.6-47.6	-	-	-
**Hardnes (°Sh)**	74	60-71.5	88.5	79.6-85.8	81-83
